# *Notes from the Field*: Suicidal Thoughts and Knowing Someone Who Died by Suicide Among Adults — United States, 2023

**DOI:** 10.15585/mmwr.mm7412a4

**Published:** 2025-04-10

**Authors:** Bhavna Singichetti, Jing Wang, Robin Lee, Michael F. Ballesteros, Karin A. Mack

**Affiliations:** ^1^Division of Injury Prevention, National Center for Injury Prevention and Control, CDC; ^2^Epidemic Intelligence Service, CDC.

**Summary**

**What is already known about this topic?**

Suicide is a leading cause of death in the United States, with approximately 49,000 deaths in 2023. Many more persons experience suicidal thoughts.

**What is added by this report?**

Using data from two probability-based online survey panels that approximate nationally representative estimates for U.S. adults, during October–November 2023 CDC estimates that more than two in five U.S. adults (42.4%) personally knew someone who died by suicide, and 5.3% of U.S. adults had suicidal thoughts during the past 12 months.

**What are the implications for public health practice?**

Suicide has a far-reaching impact on communities, and CDC recommends implementing multiple suicide prevention strategies described in CDC’s Suicide Prevention Resource for Action (e.g., lessening harms and preventing future risk). https://www.cdc.gov/suicide/resources/prevention.html

Suicide is a leading cause of death in the United States with approximately 49,000 deaths in 2023 (*1*), and many more persons seriously think about suicide (*2*). Timely data on suicidal thoughts and knowing someone who died by suicide, which can increase one’s risk for suicide (*3*), can guide public health planning and interventions.

## Investigation and Outcomes

The National Center for Health Statistics Rapid Surveys System (RSS) is an online survey based on two probability-based panels. Round 2 RSS was fielded in October–November 2023, consists of responses from 7,046 adults, and includes survey weights to approximate nationally representative estimates for U.S. adults* (*4*). Prevalences of a “Yes” response to the following questions were measured overall and by eight demographic characteristics: “At any time in the past 12 months, did you seriously think about trying to kill yourself?” and “Do you personally know anyone who has died by suicide?” ProcSurvey procedures (SAS version 9.4; SAS Institute), using the Taylor series linearization method for estimating variances, were used to calculate weighted numbers, percentages, and associated 95% CIs (*4*). These activities were reviewed by CDC, deemed not research, and conducted consistent with applicable federal law and CDC policy.^†^

Overall, 5.3% of U.S. adults reported suicidal thoughts during the past 12 months (Table). The prevalence of suicidal thoughts was higher among persons aged 18–24 and 25–44 years and persons with lower household income. Results also varied by sexual orientation, with the highest percentage among bisexual persons. Overall, 42.4% of adults reported knowing someone who died by suicide. Percentages were higher among persons aged ≥45 years, non-Hispanic White persons, veterans, persons with at least some college education, and nonmetropolitan residents. Among those who reported suicidal thoughts, more than one half (57.9%) reported knowing someone who died by suicide, compared with 41.6% among those who did not report suicidal thoughts.

**TABLE:** Numbers and percentages of persons experiencing suicidal thoughts and knowing someone who died by suicide — United States, October–November 2023*

Characteristic	Suicidal thoughts^†^ (n = 7,010)^§^		Know someone who died by suicide^¶^ (n = 6,984)^§^	
	Weighted no., thousands**	Weighted % (95% CI)**	Weighted no., thousands**	Weighted % (95% CI)**
**Overall**	**13,550**	**5.3 (4.6–5.9)**	**108,243**	**42.4 (41.0–43.8)**
**Age group, yrs**				
18–24	2,913	10.5 (7.5–13.4)	7,958	28.7 (23.8–33.6)
25–44	7,676	8.3 (7.0–9.6)	35,500	38.4 (35.9–40.9)
45–64	2,158	2.8 (2.1–3.5)	36,747	47.5 (45.1–50.0)
≥65	803	1.4 (0.7–2.1)	28,038	48.6 (46.0–51.1)
**Sex**				
Female	7,380	5.6 (4.7–6.5)	54,245	41.5 (39.5–43.5)
Male	6,170	4.9 (4.1–5.8)	53,997	43.4 (41.3–45.5)
**Sexual orientation**				
Bisexual	2,191	20.3 (14.2–26.3)	4,710	44.0 (36.8–51.2)
Gay or lesbian	543	7.9 (3.9–11.9)	3,418	49.9 (41.4–58.4)
Straight	9,016	4.1 (3.5–4.8)	91,808	42.3 (40.8–43.8)
Something else	890	18.1 (10.1–26.2)	1,791	37.0 (25.8–48.1)
Missing	910	5.7 (2.9–8.5)	6,516	41.1 (35.3–46.9)
**Race and ethnicity^††^**				
Black or African American	1,558	5.0 (3.1–6.8)	8,888	28.2 (24.4–32.1)
White	8,531	5.4 (4.6–6.2)	77,607	49.3 (47.6–51.0)
Other	817	4.0 (2.0–6.1)	7,050	35.2 (30.1–40.2)
Hispanic or Latino	2,515	5.7 (4.0–7.3)	13,637	31.1 (27.7–34.6)
**Veteran status**				
Veteran	725	3.5 (1.9–5.1)	10,078	48.8 (44.4–53.3)
Not a veteran	11,538	5.3 (4.6–6.0)	90,258	41.7 (40.1–43.2)
Missing	1,287	7.2 (4.0–10.3)	7,907	44.0 (38.2–49.8)
**Education**				
High school graduate or less	6,002	6.2 (5.0–7.4)	36,224	37.5 (35.0–40.0)
Some college	3,865	5.5 (4.4–6.7)	31,736	45.6 (43.1–48.1)
Bachelor's degree or above	3,683	4.1 (3.2–5.0)	40,282	45.3 (43.1–47.4)
**Household income, $**				
0–49,999	6,782	8.4 (7.0–9.9)	30,980	38.7 (36.1–41.4)
50,000–99,999	3,487	4.6 (3.6–5.7)	32,676	43.5 (40.9–46.1)
≥100,000	3,280	3.3 (2.5–4.1)	44,587	44.6 (42.4–46.7)
**Rural/Urban**				
Metropolitan	11,525	5.2 (4.6–5.9)	90,595	41.2 (39.7–42.7)
Nonmetropolitan	2,024	5.7 (3.9–7.6)	17,648	49.8 (46.0–53.7)
**At any time in the past 12 months, did you seriously think about trying to kill yourself?^††^**				
Yes	NA	NA	7,778	57.9 (51.5–64.4)
No	NA	NA	100,188	41.6 (40.1–43.0)

**Source:** National Center for Health Statistics, Rapid Surveys System, Round 2, October–November 2023. https://www.cdc.gov/nchs/data/series/sr_02/sr02_175.pdf

**Abbreviation:** NA = not applicable.

* All estimates included meet the National Center for Health Statistics standards of reliability.

^†^ Based on a “Yes” response to the survey question, “At any time in the past 12 months, did you seriously think about trying to kill yourself?”

^§^ Calculations are based only on responses of “Yes” and “No”. Suicidal thoughts: 36 refused or skipped the question; know someone who died by suicide: 62 refused, skipped the question, or didn’t know.

^¶^ Based on a “Yes” response to the survey question, “Do you personally know anyone who has died by suicide?”

** Nationally representative weights calibrated to the National Health Interview Survey were created to reduce coverage and nonresponse biases. Variances were estimated using the Taylor series linearization method that takes survey design into account. Weighted numbers were rounded to the nearest thousand, and weighted percentages were calculated as row percentages.

^††^ Persons identified as Hispanic or Latino (Hispanic) might be of any race. Persons identified as Black or African American, White, or Other are all non-Hispanic. Other race includes persons who identify as Asian, American Indian or Alaska Native, Middle Eastern or North African, Native Hawaiian or other Pacific Islander, or multiracial.

## Preliminary Conclusions and Actions

This investigation provides timely national estimates of and demographic variation of suicidal thoughts and knowing someone who died by suicide. This type of rapid data collection can be replicated by CDC to get the right data, in the right place, at the right time to help guide decision-making and facilitate quick action (https://www.cdc.gov/surveillance/policy-standards/data-authority.html). This study provides the latest estimates of the prevalence of knowing someone who died by suicide during one’s lifetime based on a nationally representative sample.

Results are subject to potential nonresponse bias because of the survey design (*4*); however, the finding that 5.3% of U.S. adults who reported suicidal thoughts is similar to 5.0% reported in the 2023 National Survey on Drug Use and Health (NSDUH) (*2**,**5*). Estimated percentages of persons reporting suicidal thoughts for most subgroups in RSS also aligned with NSDUH estimates. RSS has a lower response rate than other surveys conducted by CDC, and might underrepresent certain subpopulations; however, data undergo extensive quality review (*4*). The findings in this report do not infer a causal relationship; however, knowing someone who died by suicide can influence suicidal thoughts (https://www.cdc.gov/suicide/prevention/cluster.html).

This report is the first to use national survey data to indicate that more than two in five adults (42.4%) knew someone who had died by suicide, and more than one half of adults (57.9%) experiencing suicidal thoughts knew someone who had died by suicide. Identifying and supporting persons at risk for suicide, providing postvention support after a suicide occurs (i.e., activities that promote healing among survivors), and promoting safe messaging, which emphasizes that suicide is preventable, can be effective strategies and approaches in reducing suicide and future suicide risk (*3*). Strategies in CDC’s Suicide Prevention Resource for Action can normalize protective factors such as help-seeking behaviors and promoting healthy peer norms, while also reducing risk factors such as stigma about suicide and mental illness. Finally, upstream approaches including creating healthy organizational policies and protective environments, such as in places of employment and education, are also important because they can prevent a crisis point in the first place and reduce future suicide risk^§^ (*3*).

## Footnotes

* Cumulative response rates of the two panels were 3.8% and 4.0%, and Round 2 RSS overall completion rate was 37.6%.

† 45 C.F.R. part 46, 21 C.F.R. part 56; 42 U.S.C. Sect. 241(d); 5 U.S.C. Sect. 552a; 44 U.S.C. Sect. 3501 et seq.

§ For persons in crisis, help is available through the U.S. Substance Abuse and Mental Health Services Administration’s 988 Suicide and Crisis Lifeline (https://www.988lifeline.org) or by texting or calling 988.
